# Advances in Antifungal Development: Discovery of New Drugs and Drug Repurposing

**DOI:** 10.3390/ph15070787

**Published:** 2022-06-24

**Authors:** Jong H. Kim, Luisa W. Cheng, Kirkwood M. Land

**Affiliations:** 1 Foodborne Toxin Detection and Prevention Research Unit, Western Regional Research Center, USDA-ARS, 800 Buchanan St., Albany, CA 94710, USA; 2 Department of Biological Sciences, University of the Pacific, 3601 Pacific Avenue, Stockton, CA 95211, USA

This Special Issue of *Pharmaceuticals* describes recent advances accomplished in the field of antifungal development, especially the discovery of new drugs and drug repurposing. The subjects of the published articles include: new drugs from natural or synthetic sources, design, synthesis, and antifungal evaluation of newly-synthesized compounds, novel nanotechnology systems for drug delivery, drug repurposing for fungal control and/or overcoming the multidrug-resistant fungi, repurposing antifungal drugs for antiviral therapy, evaluation of differential antifungal efficacy of echinocandins, among others.

Infectious diseases caused by fungal pathogens, such as aspergillosis, candidiasis, or cryptococcus, are recurring problems. Current antifungal interventions often exhibited very limited efficacy in treating fungal infections, partly because the spectrum of the activity of conventional systemic antifungal drugs is narrow while the development of new antifungal drugs has become stagnant; azole and polyene drugs were introduced before 1980, whereas the echinocandin drug CAS was approved for the clinical uses since 2000 [1]. Besides 5-flucytosine (5FC), only three classes of antifungal drugs are used in clinical settings, namely, azoles (fluconazole (FLU), itraconazole (ITR), voriconazole (VOR), posaconazole (POS), isavuconazole (ISA)), polyenes (amphotericin B (AMB)), and echinocandins (caspofungin (CAS), micafungin (MICA), anidulafungin (ANID)).

Regarding the antifungal spectrum, for example, the yeast pathogens *Candida albicans, Candida glabrata, Candida parapsilosis,* and *Candida tropicalis* showed susceptibility to the above-mentioned antifungal drugs (AMB, 5FC, FLU, ITR, VOR, POS, ISA, CAS, MICA, and ANID), whereas the other two *Candida* species, namely, *Candida krusei* and *Candida lusitaniae*, did not show sensitivity to FLU or AMB, respectively, indicating differential susceptibilities of pathogens to the antifungal drugs exist depending on the types of fungi treated or drugs administered [2,3,4].

Also, increased incidences of pathogen resistance to conventional antifungal interventions make fungal diseases a global human health concern. For instance, sequential combination therapy is one of the strategies for effective control of fungi. However, prior exposure of fungal pathogens, such as *Candida* species or *Aspergillus fumigatus*, to azole drugs (FLU, ITR, or VOR) lowered the susceptibility of the fungal biofilms or germlings to polyenes such as AMB [5,6]. It has been determined that azole drugs could potentiate fungal tolerance to AMB via heat-shock protein 90 (Hsp90)-mediated oxidative stress defenses in pathogens [7].

Azoles are also applied in crop fields as fungicides to control plant fungal pathogens; for instance, more than 25% of current fungicide sales are azoles [8]. From the clinical perspective, increased application of agricultural fungicides provides environmental selection pressure for the development of azole-resistant fungal pathogens such as *A. fumigatus* possessing the TR34/L98H mutation, which resulted in the recent placement of azole-resistant *A. fumigatus* on the microorganism “watchlist (antibiotic resistance threat)” by the United States Centers for Disease Control and Prevention (CDC) [9].

Candidiasis is an infectious disease caused by the genus *Candida* which has been the second-highest cause of superficial/mucosal human infections and the fourth highest cause of bloodstream infections [10,11]. *Candida* species are opportunistic pathogens that change from harmless/normal inhabitants to pathogenic depending on the fluctuations in the host environment [11,12]. According to the CDC, around 95% of invasive infections by *Candida* in the United States are caused by *C. albicans*, *C. glabrata*, *C. parapsilosis*, *C. tropicalis*, and *C. krusei* [13]. While *C. albicans* is the leading cause of candidemia, increasing numbers of infections have been reported to be triggered by non-*albicans* species, which often exhibited resistance to antifungal drugs [13]. For example, around 7% of *Candida* bloodstream isolates examined at CDC showed resistance to FLU where >70% of the resistant isolates are the non-*albicans* species *C. glabrata* or *C. krusei* [13]. Therefore, the non-albicans *C. auris* or other *Candida* species have also been classified as an “urgent” or “serious” threat pathogens by CDC [9], which exhibited severe human infections (oral, vaginal, bloodstream infections) and multidrug resistance.

The toxicity triggered by conventional antifungal drugs also hampered the effectiveness of antifungal therapy. For example, the polyene AMB was the first antifungal drug introduced to clinical settings more than fifty years ago. However, AMB also triggered varying toxicity to the hosts, such as infusion-related reactions, nephrotoxicity, etc. Therefore, different types of formulations have been developed, for instance, lipid-associated AMB formulations such as the AMB lipid complex (AMB-LC), liposomal AMB (L-AMB), and colloidal dispersion of AMB (AMB-CD) [14,15].

In this Special Issue, thirteen works (seven original research articles, six reviews) were published on the recent advances in antifungal development, providing current tools, methods, strategies, or insights on antifungal therapy.

Sousa et al. [16] provided a summary of the state-of-the-art strategies for novel nanotechnology approaches for drug delivery systems and new drugs derived from natural sources. The authors provided an overview of the new drug delivery systems (types of nanoparticles) which are applicable for various antifungal agents such as azoles, amphotericin B, nystatin, etc., the drug chemical group, the route of administration (oral, topical, transdermal, etc.), and their dosage forms. The strategies not only counteract emerging fungal infections but also overcome the increase in drug-resistant strains. Of note, several mechanisms were discussed how nanoparticles overcome the development of fungal resistance [16], which include: (1) The chemical characteristics and concurrent multiple mechanisms caused by nitric oxide, chitosan, and metallic nanoparticles that destabilize fungal cell structures, (2) Packaging multiple antimicrobial drugs within the same nanoparticle, hence disrupting the resistance mechanisms, (3) Liposomes/dendrimers-based drug formulation that overcomes decreased uptake and increased efflux of drugs, and (4) Targeting antifungal drugs to the specific site of infection, enabling the localized release of high doses of drugs while keeping the total concentrations of the drugs administered low. The review [16] also focused on the identification of new natural antifungal compounds from the marine environment; the approaches provide platforms to discover possible new drug leads while highlighting the challenges of the translation of the identified natural compounds into the clinical pipeline.

Bouz and Doležal [17] reviewed recent advances in antifungal drug development in clinical settings. The review summarized novel antifungal agents in clinical development including first-in-class agents, new chemical structures developed against established molecular targets, modifications of formulation to marketed antifungal drugs, repurposing of non-antifungal agents to treat fungal infections, and membrane interacting peptides as antifungals, among others. Of note, the review described further the immunotherapy using the antifungal antibodies MAb 2G8 and efungumab (mycograb) advancing into clinical trials. MAb 2G8 targets laminarin (consisting mainly of β-glucans) and thus binds to the cell walls of *Candida albicans* and *Cryptococcus neoformans*, inhibiting fungal growth and capsule formation [18], whereas the new, humanized version of monoclonal MAb 2G8 precisely targets β-1,3 glucans of *Candida* spp. including *Candida auris* [19]. Novel antifungal targets are also discussed, such as heme biosynthesis and sphingolipid synthesis which are unique fungal target pathways, thus enabling the designing of novel drugs and overcoming the pathogen resistance to conventional antifungal interventions.

Recently, β-glucans, a subgroup of polysaccharides, have been implicated in medicinal applications, such as the treatment of diabetes and immunomodulation [20]. Since β-glucans often exhibit very limited water solubility, the cationization, namely, the addition of a positive charge to the polymer structures using “glycidyl trimethyl ammonium chloride (GTMAC)” has been a tool to enhance water solubility. Meanwhile, the interaction between the positively charged polymers, namely, polycations, and a biological membrane could be the cause of the toxicity exerted by the developed macromolecules, hence it became a very attractive strategy to improve the antifungal activity of the candidate polymers. In their research article, Kaminski et al. [21] investigated cationic derivatives of natural β-glucan polymers that were synthesized by reacting the polysaccharides from *Saccharomyces boulardii* (SB) and *Cetraria islandica* with GTMAC. In the study, three different polymer structures were obtained exhibiting selective antifungal activities against *Scopulariopsis brevicaulis*, *Aspergillus brasiliensis*, and *Fusarium solani*. In an in vivo model study of *Aspergillus brasiliensis* infection in *Galleria mellonella* (insect) using SB derivative, positive antifungal results were also observed while toxicity assay via fibroblast 3T3-L1 cell line revealed negligible toxicity of the compounds at various concentrations.

Using the fission yeast *Schizosaccharomyces pombe* (a model fungal system), Yagüe et al. [22] determined that various echinocandin drugs induce differential effects in cytokinesis progression and cell integrity in fungi. *S. pombe* contains three β(1,3)-D-glucan synthases (GSs), Bgs1, Bgs3, and Bgs4, which exert essential functions with non-overlapping roles involved in maintaining cell integrity and morphogenesis. Results by Yagüe et al. demonstrated that MICA exerts fungicidal activity by solely inhibiting Bgs4, whereas lethal doses of other echinocandin drugs ANID and CAS trigger an early cytokinesis arrest during fungal growth possibly by the concerted inhibition of several GSs.

Clotrimazole has been used for treating the yeast pathogen *C. albicans* and other fungi. The antifungal mechanism of clotrimazole is to interfere with ergosterol biosynthesis via the inhibition of the fungal cytochrome 14α-demethylase, which eventually triggers the disruption of the structure/function of the fungal cell wall including cell wall leakage. Since clotrimazole has been applied for treating vulvovaginal candidiasis for around 50 years, Mendling et al. [23], via analyzing thirty-seven randomized controlled studies, tried to estimate the current effectiveness of topical clotrimazole under different disease severity of populations infected. In patients with uncomplicated vulvovaginal candidiasis, application of single intravaginal doses of 500 mg clotrimazole (vaginal tablets) resulted in high cure rates, thus it was concluded that a single dose of clotrimazole 500 mg was equally effective as multiple doses of lower clotrimazole strengths. Moreover, prolonged treatment with the drug showed effectiveness in severe, recurrent infections and further in pregnant women. Therefore, Mendling et al. concluded that, despite its long-term usage, clotrimazole resistance in vaginal candidosis is scarce and hence, clotrimazole can continuously be used for vaginal health in the future [23].

*Cryptococcus neoformans* is also an opportunistic yeast pathogen causing cryptococcus, especially in the immune-compromised patients; around 73% of 223,100 annual cases of cryptococcal meningitis are diagnosed in sub-Saharan Africa with a 75% mortality rate [24]. Since photodynamic treatment (PDT) is a method of curing aerobic microbes susceptible to oxidative damage [25], Ogundeji et al. [24] investigated the photodynamic activity of acetylsalicylic acid (ASA; aspirin) against respiring cryptococcal cells. Cryptococcal cells were exposed to 0.5 or 1 mM of ASA with ultraviolet light (UVL) for 10 min, which resulted in a significant reduction in the fungal growth compared to the non-treated control cells. The treated cells exhibited: (a) a significant loss of mitochondrial membrane potential, (b) cellular accumulation of toxic reactive oxygen species, (c) altered ultrastructural appearance, and (d) limited expression levels of the capsular-associated gene, *CAP64*, leading to an acapsular phenotype.

Retinoids are chemical compounds derived from vitamin A or its structural analogs including all-*trans* retinoic acid (ATRA; tretinoin), a vitamin A metabolite. Retinoids possess a potent antimicrobial activity against a broad spectrum of pathogens, including bacteria, fungi and viruses. In their review, Cosio et al. [26] evaluated/summarized the results of thirty-nine in vitro/in vivo antifungal studies that demonstrated the antifungal efficacy of retinoids against human opportunistic fungal pathogens, such as yeast fungi (*Candida* spp., *Rhodotorula mucilaginosa*, *Malassezia furfur*) colonizing on the skin/mucosal surfaces of humans, environmental molds (*Aspergillus* spp., *Fonsecae monofora*) and several species of dermatophytes infecting humans and animals. The data from retinoid studies indicated that vitamin A or ATRA lowers the incidence and severity of fungal diseases via the immunoadjuvant properties of these compounds, where the antifungal efficacy, tolerability, and safety profile of test compounds have been proven against localized and systemic fungal infections [26].

In the study by Rodríguez-Villar et al. [27], a set of chemical compounds were synthesized and their antifungal activities were examined against *C. albicans*, *C. glabrata*, and *C. tropicalis* strains. They found that chemical derivatives containing “3-phenyl-1H-indazole” moiety exhibited the highest, broad anticandidal activity. In particular, *N*,*N*-diethylcarboxamide substituent showed the highest activity against *C. albicans* and *C. glabrata* (both miconazole susceptible and resistant species). Collectively, results indicated that the 3-phenyl-1H-indazole moiety can be a useful scaffold for the development of new anticandidal drugs.

Azole fungicides bind to and inhibit lanosterol 14α-demethylase, which is a fungal cytochrome P450 (CYP) enzyme involved in the ergosterol biosynthesis in fungal pathogens. While azoles are considered the most successful class of drugs for fungal control, extensive use of azole-class fungicides and prophylactic antifungal therapy resulted in the development of fungal resistance to clinical azole drugs. In their review, Kane and Carter [28] documented the enhancement of the potency of azole drugs with drug synergy, where significant antifungal synergism between azoles (FLU, ITR, VOR, ISA, POS, Ketoconazole, Miconazole) and known chemicals such as other antifungals (polyenes, echinocandins, etc.), anti-bacterials, anti-parasitics, anti-virals, calcium inhibitors, statins, bisphosphonates, immunomodulators, psychoactives, calcineurine inhibitors, essential oil extracts, etc., has been evaluated. Furthermore, azole synergy with novel compounds was also presented where the synergism with heat shock protein 90 (Hsp90) inhibitors, isoquinolone, phthalazine, natural metabolites berberine, piperidol, caffeic acid, guttiferone, the anti-inflammatory celecoxib, and phenylpentanol β-glucan synthase inhibitor SCY-078, ion chelator DIBI, target of rapamycin (TOR) inhibitor AZD8055, efflux modulators, as well as novel antifungal chalcones effectively control fungal pathogens including azole resistant *C. albicans*, *C. glabrata* and other *Candida* species. Considering no azole-based antifungal combinations are currently applied to treat fungal infections, co-application of secondary compounds exhibiting azole synergy, as shown above, could function as new fungal control regimens. It was concluded that “azole synergy” with “secondary compounds” will be a promising strategy for treating intrinsic/acquired resistance to the conventional drugs exerted by certain fungal pathogens [28].

The wide distribution of feline sporotrichosis in Brazil has been problematic, requiring new therapeutic alternatives [29]. In the in vitro and in silico analyses of new synthetic derivatives against wild type and non-wild type *Sporothrix brasiliensis*, de Souza et al. [29] demonstrated the antifungal efficacy of derivatives of three novel hydrazone and eleven novel quinone using the broth microdilution method, where the minimum inhibitory and fungicidal concentrations of test compounds ranged from 1 to >128 μg/mL. Particularly, the three hydrazone derivatives were determined as promising antifungal candidates against ITR-resistant *S. brasiliensis*, which is the most virulent and prevalent species causing feline sporotrichosis [29].

In view of the difficulties to treat invasive fungal infections, the development of new, alternative antifungal strategies that are efficient and appropriate measures are urgently needed. Antifungal drug repurposing is the repositioning process of already marketed medicinal drugs developed for treating human diseases to cure fungal infections [30]. The key merit of drug repurposing is that the mechanisms of action, cellular targets, or safety of the marketed drugs are already known, thus enabling accelerated regulatory approval once antifungal efficacy is determined. In their review, Peyclit et al. [31] provided a comprehensive summary of current antifungal drugs, their mechanisms of resistance, as well as an overview of the potent antifungal activity of non-traditional antifungal drugs. The review also presented mechanisms of action and the synergistic drug/compound combinations that enhance the efficacy of current antifungal interventions. Notably, drug repurposing for treating the emerging multidrug resistant (MDR) fungus such as *Candida auris*, *Aspergillus* or *Cryptococcus* species has been discussed, addressing the potential of antifungal drug repurposing to cure the difficult human fungal pathogens [31].

Conversely, certain antifungal drugs have been repurposed to treat viral or amoeboidal infections. Schloer et al. in their review [32] postulated that repurposing marketed antifungal drugs with well-known safety profiles can additionally target “host cell factors” necessary for viral proliferation, such as virus entry, replication, etc. Hence, these host cell factors could be a promising cellular target for the development of novel prophylaxis and treatment approaches against viral infections. ITR is one of the azoles inhibiting fungal ergosterol biosynthesis. Studies have shown that, in host cells, ITR directly interacts with the endolysosomal cholesterol transporter “Niemann–Pick disease, type c1 (npc1)” protein. A dysfunctional NPC1 interferes with the intracellular lipid transport, thus resulting in the excessive accumulation of lipids such as cholesterol in the endolysosomal compartment [33]. Importantly, NPC1 is recently explored as an antiviral drug target that can be interfered with ITR [34,35]. Other host cell factors to be interfered with ITR for the treatment of viral infections includes oxysterolbinding protein 1 (OSBP) and OSBP-Related Proteins (ORP) involved in lipid homeostasis [36], mammalian target of rapamycin (mTOR), hedgehog, and Wnt signaling pathways that regulate apoptosis and/or counteracts stress-induced autophagy [37,38], etc.

*Acanthamoeba castellanii* is a causative agent (amoebaoid) of *Acanthamoeba* keratitis (AK), a serious eye infection resulting in an inflammation in the cornea, which could trigger permanent visual impairment/blindness [39]. Isavuconazonium sulfate is an FDA-approved antifungal drug for treating invasive fungal diseases such as aspergillosis and mucormycosis. The prodrug isavuconazonium sulfate is metabolized further into the active molecule ISA which previously exhibited amebicidal and cysticidal activity against *Acanthamoeba* T4 strains. However, the activity of the prodrug isavuconazonium sulfate against trophozoites and cysts was not determined. Shing et al. [39] recently demonstrated that isavuconazonium also possessed potent amoebicidal activity at as low as 1.4 nM and prevented excystation of cysts at as low as 136 μM, thus suggesting that, like ISA, the prodrug isavuconazonium maintains activity against *Acanthamoeba* T4 strains, which could be adapted for *Acanthamoeba* keratitis treatment.

In summary, the research articles and reviews presented in this Special Issue provide useful information and insight for the development of new antifungal drugs or intervention strategies. Identification of new, safe molecules, and cellular targets, as well as elucidation of their antifungal mechanisms of action will further the effective control of fungal pathogens, especially those resistant to current therapeutic agents.

## References

[1] Roemer T., Krysan D.J. (2014). Antifungal Drug Development: Challenges, Unmet Clinical Needs, and New Approaches. Cold Spring Harb. Perspect. Med..

[2] Houšť J., Spížek J., Havlíček V. (2020). Antifungal Drugs. Metabolites.

[3] Nami S., Aghebati-Maleki A., Morovati H., Aghebati-Maleki L. (2019). Current antifungal drugs and immunotherapeutic approaches as promising strategies to treatment of fungal diseases. Biomed. Pharmacother..

[4] Nett J.E., Andes D.R. (2016). Antifungal Agents: Spectrum of Activity, Pharmacology, and Clinical Indications. Infect. Dis. Clin. N. Am..

[5] Rajendran R., Mowat E., Jones B., Williams C., Ramage G. (2015). Prior in vitro exposure to voriconazole confers resistance to amphotericin B in *Aspergillus fumigatus* biofilms. Int. J. Antimicrob. Agents.

[6] Vazquez J.A., Arganoza M.T., Boikov D., Yoon S., Sobel J.D., Akins R.A. (1998). Stable Phenotypic Resistance of *Candida* Species to Amphotericin B Conferred by Preexposure to Subinhibitory Levels of Azoles. J. Clin. Microbiol..

[7] Delattin N., Cammue B.P., Thevissen K. (2014). Reactive oxygen species-inducing antifungal agents and their activity against fungal biofilms. Future Med. Chem..

[8] Bowyer P., Denning D.W. (2014). Environmental fungicides and triazole resistance in *Aspergillus*. Pest Manag. Sci..

[9] Centers for Disease Control and Prevention (U.S.) (2019). Antibiotic Resistance Threats in the United States, 2019.

[10] Bongomin F., Gago S., Oladele R.O., Denning D.W. (2017). Global and Multi-National Prevalence of Fungal Diseases—Estimate Precision. J. Fungi.

[11] Brown G.D., Denning D.W., Gow N.A.R., Levitz S.M., Netea M.G., White T.C. (2012). Hidden Killers: Human Fungal Infections. Sci. Transl. Med..

[12] Gonçalves B., Ferreira C., Alves C.T., Henriques M., Azeredo J., Silva S. (2016). Vulvovaginal candidiasis: Epidemiology, microbiology and risk factors. Crit. Rev. Microbiol..

[13] Centers for Disease Control and Prevention (U.S.) Invasive Candidiasis Statistics. https://www.cdc.gov/fungal/diseases/candidiasis/invasive/statistics.html.

[14] Hamill R.J. (2013). Amphotericin B Formulations: A Comparative Review of Efficacy and Toxicity. Drugs.

[15] Marena G.D., Ramos M.A.d.S., Bauab T.M., Chorilli M. (2022). A Critical Review of Analytical Methods for Quantification of Amphotericin B in Biological Samples and Pharmaceutical Formulations. Crit. Rev. Anal. Chem..

[16] Sousa F., Ferreira D., Reis S., Costa P. (2020). Current Insights on Antifungal Therapy: Novel Nanotechnology Approaches for Drug Delivery Systems and New Drugs from Natural Sources. Pharmaceuticals.

[17] Bouz G., Doležal M. (2021). Advances in Antifungal Drug Development: An Up-to-Date Mini Review. Pharmaceuticals.

[18] Rachini A., Pietrella D., Lupo P., Torosantucci A., Chiani P., Bromuro C., Proietti C., Bistoni F., Cassone A., Vecchiarelli A. (2007). An Anti-Beta-Glucan Monoclonal Antibody Inhibits Growth and Capsule Formation of *Cryptococcus neoformans* In Vitro and Exerts Therapeutic, Anticryptococcal Activity In Vivo. Infect. Immun..

[19] Di Mambro T., Vanzolini T., Bruscolini P., Perez-Gaviro S., Marra E., Roscilli G., Bianchi M., Fraternale A., Schiavano G.F., Canonico B. (2021). A new humanized antibody is effective against pathogenic fungi in vitro. Sci. Rep..

[20] Vetvicka V., Vannucci L., Sima P., Richter J. (2019). Beta Glucan: Supplement or Drug? From Laboratory to Clinical Trials. Molecules.

[21] Kaminski K., Skora M., Krzyściak P., Stączek S., Zdybicka-Barabas A., Cytryńska M. (2021). Synthesis and Study of Antifungal Properties of New Cationic Beta-Glucan Derivatives. Pharmaceuticals.

[22] Yagüe N., Gómez-Delgado L., Curto M.Á., Carvalho V.S.D., Moreno M.B., Pérez P., Ribas J.C., Cortés J.C.G. (2021). Echinocandin Drugs Induce Differential Effects in Cytokinesis Progression and Cell Integrity. Pharmaceuticals.

[23] Mendling W., Atef El Shazly M., Zhang L. (2020). Clotrimazole for Vulvovaginal Candidosis: More Than 45 Years of Clinical Experience. Pharmaceuticals.

[24] Ogundeji A.O., Mjokane N., Folorunso O.S., Pohl C.H., Nyaga M.M., Sebolai O.M. (2021). The Repurposing of Acetylsalicylic Acid as a Photosensitiser to Inactivate the Growth of Cryptococcal Cells. Pharmaceuticals.

[25] Stapleton M., Rhodes L.E. (2003). Photosensitizers for photodynamic therapy of cutaneous disease. J. Dermatol. Treat..

[26] Cosio T., Gaziano R., Zuccari G., Costanza G., Grelli S., Di Francesco P., Bianchi L., Campione E. (2021). Retinoids in Fungal Infections: From Bench to Bedside. Pharmaceuticals.

[27] Rodríguez-Villar K., Hernández-Campos A., Yépez-Mulia L., Sainz-Espuñes T.d.R., Soria-Arteche O., Palacios-Espinosa J.F., Cortés-Benítez F., Leyte-Lugo M., Varela-Petrissans B., Quintana-Salazar E.A. (2021). Design, Synthesis and Anticandidal Evaluation of Indazole and Pyrazole Derivatives. Pharmaceuticals.

[28] Kane A., Carter D.A. (2022). Augmenting Azoles with Drug Synergy to Expand the Antifungal Toolbox. Pharmaceuticals.

[29] de Souza L.C.d.S.V., Alcântara L.M., de Macêdo-Sales P.A., Reis N.F., de Oliveira D.S., Machado R.L.D., Geraldo R.B., dos Santos A.L.S., Ferreira V.F., Gonzaga D.T.G. (2022). Synthetic Derivatives against Wild-Type and Non-Wild-Type *Sporothrix brasiliensis*: In Vitro and In Silico Analyses. Pharmaceuticals.

[30] Cha Y., Erez T., Reynolds I.J., Kumar D., Ross J., Koytiger G., Kusko R., Zeskind B., Risso S., Kagan E. (2018). Drug repurposing from the perspective of pharmaceutical companies. Br. J. Pharmacol..

[31] Peyclit L., Yousfi H., Rolain J.-M., Bittar F. (2021). Drug Repurposing in Medical Mycology: Identification of Compounds as Potential Antifungals to Overcome the Emergence of Multidrug-Resistant Fungi. Pharmaceuticals.

[32] Schloer S., Goretzko J., Rescher U. (2022). Repurposing Antifungals for Host-Directed Antiviral Therapy?. Pharmaceuticals.

[33] Vanier M., Millat G. (2003). Niemann–Pick disease type C. Clin. Genet..

[34] Côté M., Misasi J., Ren T., Bruchez A., Lee K., Filone C.M., Hensley L., Li Q., Ory D., Chandran K. (2011). Small molecule inhibitors reveal Niemann–Pick C1 is essential for Ebola virus infection. Nature.

[35] Herbert A.S., Davidson C., Kuehne A.I., Bakken R., Braigen S.Z., Gunn K.E., Whelan S.P., Brummelkamp T.R., Twenhafel N.A., Chandran K. (2015). Niemann-Pick C1 Is Essential for Ebolavirus Replication and Pathogenesis In Vivo. mBio.

[36] Schloer S., Goretzko J., Kühnl A., Brunotte L., Ludwig S., Rescher U. (2019). The clinically licensed antifungal drug itraconazole inhibits influenza virus in vitro and in vivo. Emerg. Microbes Infect..

[37] Karam B.S., Morris R.S., Bramante C.T., Puskarich M., Zolfaghari E.J., Lotfi-Emran S., Ingraham N.E., Charles A., Odde D.J., Tignanelli C.J. (2021). mTOR inhibition in COVID-19: A commentary and review of efficacy in RNA viruses. J. Med. Virol..

[38] Kuss-Duerkop S.K., Wang J., Mena I., White K., Metreveli G., Sakthivel R., Mata M.A., Muñoz-Moreno R., Chen X., Krammer F. (2017). Influenza virus differentially activates mTORC1 and mTORC2 signaling to maximize late stage replication. PLoS Pathog..

[39] Shing B., Balen M., Debnath A. (2021). Evaluation of Amebicidal and Cysticidal Activities of Antifungal Drug Isavuconazonium Sulfate against Acanthamoeba T4 Strains. Pharmaceuticals.

